# Modification of magnetic mesoporous N-doped silica nanospheres by CuO NPs: a highly efficient catalyst for the multicomponent synthesis of some propellane indeno indole derivatives†

**DOI:** 10.1039/d2ra06221f

**Published:** 2022-12-06

**Authors:** Mina Zare, Leila Moradi

**Affiliations:** Department of Organic Chemistry, Faculty of Chemistry, University of Kashan P.O. Box 8731753153 Kashan Iran l_moradi@kashanu.ac.ir +98-3155912336

## Abstract

Herein, magnetic mesoporous N-doped silica nanospheres decorated by CuO nanoparticles (M-MNS/CuO) were prepared and used for the green and efficient synthesis of some [3.3.3] propellane indeno[1,2-*b*] indole derivatives. In order to prepare N-doped silica nanoparticles, tetraethyl orthosilicate (TEOS) was used as the silica source, and diethanolamine (DEA) as a nitrogen precursor. Immobilization of CuO nanoparticles on the mesoporous N-doped silica nanosphere surfaces increases the surface area of catalyst and provides Lewis acidic sites in addition to nitrogen atoms as active basic sites. The presence of nitrogen atoms and copper oxide nanoparticles in the catalyst structure, give dual acidic and basic properties. The synthesized catalyst was characterized by FESEM, EDS, HRTEM, XRD, VSM, FTIR, and BET techniques which proved its magnetic core shell structure.

## Abstract 2

Herein, magnetic mesoporous N-doped silica nanospheres decorated by CuO nanoparticles (M-MNS/CuO) were prepared and used for the green and efficient synthesis of some [3.3.3] propellane indeno[1,2-*b*] indole derivatives.

## Graphical abstract

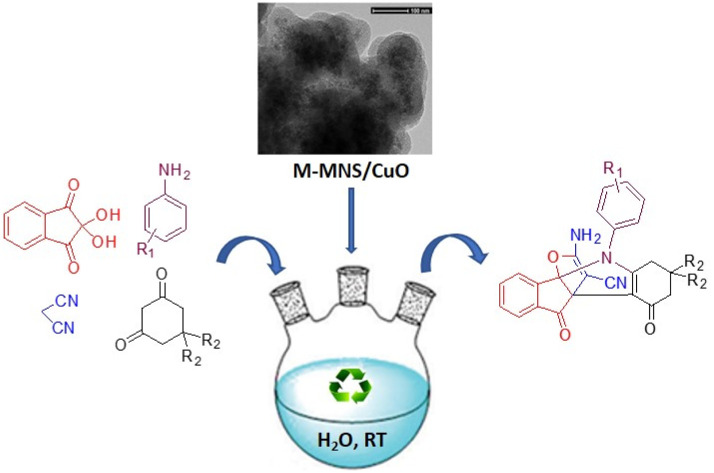

## Introduction

**Fig. 1:**
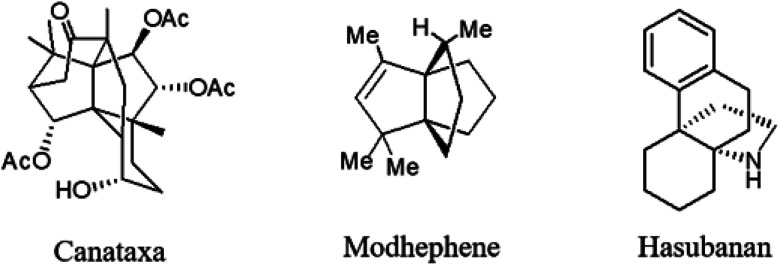
Natural products containing tricyclic propellanes.

Propellanes as annulated tricyclic systems with a common carbon–carbon covalent bond, have many applications in synthesis of bioactive medicinal compounds^1^ or polymers.^2^ These tricyclic systems are part of many natural products, such as modhephene 1,^3^ canataxa 2 (ref. 4) and hasubanan alkaloids 3 (ref. 5) (Fig. 1).

Also, the chemical and biological activity of propellanes has turned them into attractive structures.^6–13^ Some domino multicomponent reactions for the preparation of [3.3.3] propellanes have been reported.

“Domino reactions” have received a great attention in recent years as an efficient synthetic methodology for the construction of structurally complex molecules, starting from simple materials. The domino reaction is a time-resolved process in which two or more bond-forming reactions occur under similar conditions and subsequent transformation takes place at the functionalities obtained in the former transformation. They have several inherent advantages including bond forming as well as time and cost efficiency, atom economy, environmental friendliness as well as applicability to diversity-oriented high-throughput synthesis and combinatorial chemistry in the form of multicomponent transformations. The development of sequences that combine transformations of different fundamental mechanisms, broadens the scope of their application in synthetic chemistry. In 2012, Alizadeh and his co-workers have successfully reported the catalyst free synthesis of the heterocyclic [3.3.3] propellanes by the domino four-component reactions.^3^ In 2013 Some of heterocyclic propellanes were prepared using Et_3_N in ethanol by Yan *et al.*^14^ Due to the limitations of homogeneous catalysts, heterogeneous catalysts were used to synthesis of propellane indeno indoles. In 2017, these compounds were prepared using CSB-Ni(ii) as a heterogeneous catalyst^15^ and in 2021, the hollow mesoporous boron nitride spheres decorated by CuO was successfully prepared and used as an efficient catalyst for the synthesis of [3.3.3] propellane indeno[1,2-*b*] indole derivatives.^16^ Nowadays, nano catalysts (or nano based heterogeneous catalysts), as highly efficient, green, reusable with high surface area, were used in chemical synthesis and industrial applications.^17–21^ Among the nano catalysts, core–shell structures has attracted more attentions. Between the core–shell structures, magnetic core with silica shell structures, have more applications as catalyst support, drug delivery, chromatography and biological sensors due to their easy separation, high surface area, narrow pore size distribution and thermal stability.^22–27^ Nitrogen doping in mesoporous silica frameworks, can improved their catalytic activity and also the chemically attachment of organic or inorganic groups on the N-doped silica frameworks can be performed easier.^28–33^ In 2020 N-doping of the magnetic hollow mesoporous silica rods was performed using diethanol amine (DEA) as nitrogen precursor. The prepared heterogeneous basic catalyst was used for the synthesis of some dihydropyridine derivatives under green conditions.^25^ Also core–shell magnetic mesoporous N-doped silica nanoparticles as a solid base catalysts was used for the preparation of some arylpyrimido[4,5-b]quinoline diones.^34^

**Scheme 1:**
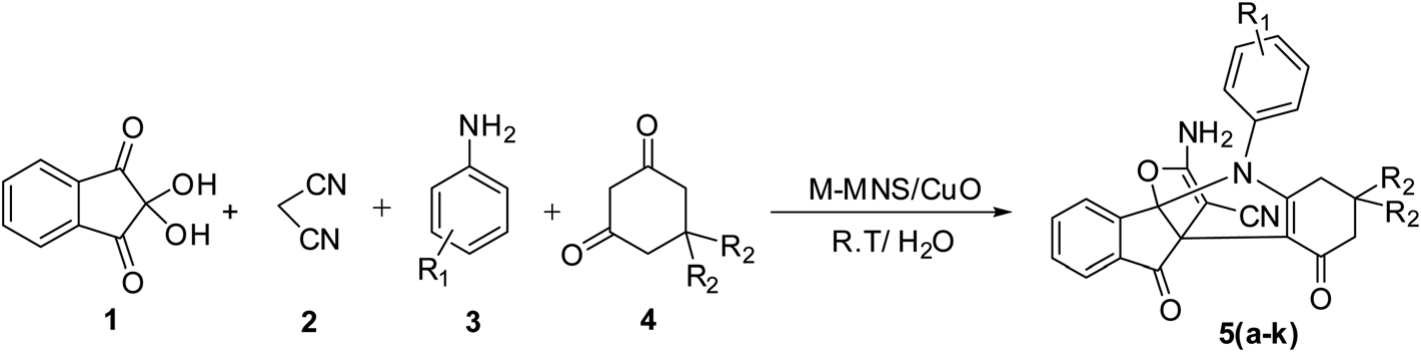
Synthesis of [3.3.3] propellane indeno[1,2-*b*] indole derivatives in the presence of M-MNS/CuO.

In this study, we try to prepare a magnetic mesoporous N-doped silica nanospheres decorated by CuO (M-MNS/CuO) as an effective catalyst for the synthesis of some propellane derivatives. Doping of nitrogen in silica framework was done using diethanolamine (as nitrogen precursor) (Scheme 1).

## Experimental section

### Materials and instrumentation

Chemicals were purchased from Sigma-Aldrich and Merck companies. The BET analysis was applied to determine the specific surface area of catalysts (Tristar 3000, Micromeritics). Crystal structure of catalyst was evaluated by X-ray diffraction using Philips X'Pert MPD diffractometer at the 2*θ* range of 10–80°. VSM analyzer was used for determining the magnetic property by Meghnatis Daghigh Kavir Company-Iran. The morphology of prepared catalyst was observed using FESEM with a scanning electron electrode operating at 15 kV. To examine the exact morphology of the catalyst structure TEM on a FEI Tecnai F20 USA microscope at an accelerating voltage of 200 kV was used. Characterization of products was done by ^1^H NMR and ^13^C NMR methods, in the DMSO-*d*_6_ as a solvent on a Bruker DRX-400 spectrometer. FT-IR spectrum was recorded in a spectrophotometer (PerkinElmer 781) to determine the functional groups of propellane indeno indoles and M-MNS/CuO.

### Synthesis of NiFe_2_O_4_ nanoparticles

**Scheme 2:**
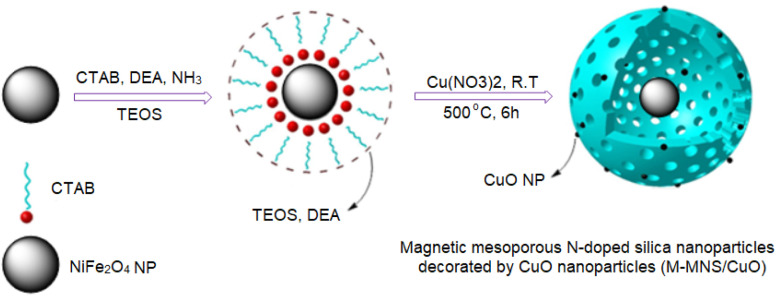
Synthetic process of M-MNS/CuO.

2 mmol FeCl_3_·6H_2_O and 1 mmol NiCl_2_·6H_2_O were added to 30 ml ethylene glycol under vigorously stirring. Then, 20 mmol NaOH was added to above solution. After 30 min, obtained mixture was placed to the Teflon autoclave for 24 h at 200 °C. Finally, the obtained black magnetic product was separated with a strong magnet and dried (Scheme 2).

### Synthesis of magnetic mesoporous N-doped silica nanospheres decorated by CuO nanoparticles (M-MNS/CuO)

0.1 g NiFe_2_O_4_ and 0.7 g CTAB were added to 120 ml ethanol/H_2_O (volume ratio of 2 : 1) and sonicated for 20 min. Then, 1 ml ammonium solution 25% and 1 ml diethanolamine were added and the obtained mixture was stirred at room temperature. After that, 0.4 ml TEOS in 10 ml ethanol was added to the above mixture and stirring was continued for 24 h. After the time, obtained product was separated with a strong magnet, washed with EtOH and distilled water and dried for 8 h at 80 °C. To incorporate CuO nanoparticles on the surfaces of M-MNs, 0.45 mmol (0.084 g) Cu(NO_3_)_2_ dissolved in 10 ml DI water was added drop by drop to 0.5 g of M-MNS in 5 ml water and stirred for 3 h. The obtained product was separated by magnet, washed with ethanol and H_2_O and dried. In final step, calcination at 500 °C for 6 h produced the N-MNS/CuO (Scheme 2).

### Synthesis of [3.3.3] propellane indeno[1,2-*b*] indole derivatives in the presence of M-MNS/CuO

1 mmol diketone and 1 mmol aniline were reacted to gather in 3 ml distilled water at 70 °C in the presence of 0.006 g catalyst. Then 1 mmol ninhydrine, 1 mmol malononitrile and 0.006 g catalyst were reacted in 3 ml distilled water at 70 °C. Two above mixtures were added together and stirred for appropriate time at room temperature (the reaction process was monitored by TLC). After the completion of reaction, magnetic catalyst was separated from the reaction mixture with strong magnet. Then, the mixture was filtrate to separate the product and washing the obtained compound with EtOH and DI water are final steps of the synthetic procedure. All of products were characterized and spectral data were consistent in comparison with authentic samples.^14–16^

### Spectral data

#### 12-Amino-5-(4-boromophenyl)-9,10-dioxo-5,6,7,8,9,10-hexahydro-4*b*,9*b*-(epoxyetheno)indeno[1,2-*b*]indole-11-carbonitrile (5a)

Mp: 227–229;^14^ FTIR (KBr) (*v*_max_/cm^−1^): 3401, 3316, 3197, 2945, 2191, 1747,1707, 1674, 1643, 1591, 1492, 1459, 1420, 1351, 1311, 1265, 1219, 1196, 1074, 1011, 920, 881, 841, 764, 657; ^1^H NMR (DMSO-*d*_6_, 400 MHz) *δ* (ppm): 8.90 (s, 1H, NH), 7.87 (d, *J* = 7.8 Hz, 1H, ArH), 7.76 (t, *J* = 9.1 Hz, 3H, ArH), 7.65 (t, *J* = 7.5 Hz, 1H, ArH), 7.27 (d, *J* = 8.1 Hz, 2H, ArH), 6.90 (d, *J* = 7.8 Hz, 1H, ArH), 5.91 (s, 1H, NH), 2.43 (m, 1H, CH), 2.26–2.11 (m, 2H, CH_2_), 2.07–1.99 (m, 1H, CH), 1.88–1.78 (m, 2H, CH_2_).

#### 12-Amino-5-(4-chlorophenyl)-9,10-dioxo-5,6,7,8,9,10-hexahydro-4*b*,9*b*-(epoxyetheno)indeno[1,2-*b*]indole-11-carbonitrile (5b)

Mp: 235–237 °C;^14^ FTIR (KBr) (*v*_max_/cm^−1^): 3402, 3316, 2923, 2191, 1716, 1672, 1643, 1597, 1542, 1493, 1457, 1410, 1349, 1262, 1196, 1147, 1088, 1012, 920, 852; ^1^H NMR (DMSO-*d*_6_, 400 MHz) *δ* (ppm): 8.89 (s, 1H, NH), 8.03–7.98 (m, 1H, ArH), 7.87 (d, *J* = 7.6 Hz, 1H, ArH), 7.77 (t, *J* = 7.5 Hz, 1H, ArH), 7.67–7.61 (m, 3H, ArH), 7.34 (d, *J* = 8.2 Hz, 1H, ArH), 6.90 (d, *J* = 7.8 Hz, 1H, ArH), 5.91 (s, 1H, NH), 2.24–2.12 (m, 2H, CH_2_), 2.06–1.96 (m, 2H, CH_2_), 1.84–1.80 (m, 2H, CH_2_).

#### 12-Amino-5-(4-methoxyphenyl)-9,10-dioxo-5,6,7,8,9,10-hexahydro-4*b*,9*b*-(epoxyetheno)indeno[1,2-*b*]indole-11-carbonitrile (5c)

Mp: 223–225;^14^ FTIR (KBr) (*v*_max_/cm^−1^): 3404, 3318, 3198, 2944, 2192, 1707, 1674, 1543, 1592, 1510, 1461, 1411, 1350, 1310, 1260, 1220, 1196, 1148, 1074, 1012, 920, 843, 767, 658; ^1^H NMR (DMSO-*d*_6_, 400 MHz) *δ* (ppm): 8.70 (s, 1H, NH), 7.86 (d, *J* = 7.8 Hz, 1H, ArH), 7.76 (t, *J* = 7.5 Hz, 1H, ArH), 7.64 (t, *J* = 7.4 Hz, 1H, ArH), 7.20 (d, *J* = 8.5, 2H, ArH), 7.08 (d, *J* = 8.6 Hz, 2H, ArH), 6.95 (d, *J* = 7.8 Hz, 1H, ArH), 5.90 (s, 1H, NH), 3.85 (s, 3H, CH_3_), 2.41–2.34 (m, 2H, CH_2_), 2.18–2.15 (m, 2H, CH_2_), 2.01–1.97 (m, 2H, CH_2_).

#### 12-Amino-5-(*P*-tolylphenyl)-9,10-dioxo-5,6,7,8,9,10-hexahydro-4*b*,9*b*-(epoxyetheno)indeno[1,2-*b*]indole-11-carbonitrile (5d)

Mp: 221–223 °C;^14^ FTIR (KBr) (*v*_max_/cm^−1^): 3401, 3316, 3198, 2947, 2768, 2191, 1747, 1708, 1674, 1642, 1592, 1540, 1510, 1461, 1417, 1350, 1264, 1220, 1196, 1147, 1074, 1011, 921, 847, 766; ^1^H NMR (DMSO-*d*_6_, 400 MHz): *δ* (ppm): 8.76 (s, 1H, NH), 7.87 (d, *J* = 7.6 Hz, 2H, ArH), 7.74 (t, *J* = 7.5 Hz, 1H, ArH), 7.34 (d, *J* = 8.0 Hz, 2H, ArH), 7.17 (d, *J* = 7.9 Hz, 2H, ArH), 6.90 (d, *J* = 7.8 Hz, 1H, ArH), 5.91 (s, 1H, NH), 2.70 (t, *J* = 6.3 Hz, 2H, CH_2_), 2.40 (s, 3H, CH_3_), 2.27 (t, *J* = 6.5 Hz, 2H, CH_2_), 1.95 (t, *J* = 6.7 Hz, 2H, CH_2_).

#### 12-Amino-5-(4-iodophenyl)-9,10-dioxo-5,6,7,8,9,10-hexahydro-4*b*,9*b*-(epoxyetheno)indeno[1,2-*b*]indole-11-carbonitrile (5e)

Mp: 226–228 °C;^15^ FTIR (KBr) (*v*_max_/cm^−1^): 3403, 3317, 2925, 2191, 1708, 1673, 1642, 1593, 1537, 1488, 1457, 1419, 1350, 1264, 1219, 1196, 1146, 1074, 1008, 920, 844, 767, 656; ^1^H NMR (DMSO-*d*_6_, 400 MHz): *δ* (ppm): 8.89 (s, 1H, NH), 8.01 (d, *J* = 7.6 Hz, 1H, ArH), 7.92–7.86 (m, 3H, ArH), 7.77 (t, *J* = 7.6 Hz, 1H, ArH), 7.65 (t, *J* = 6.5 Hz, 1H), 7.11 (d, *J* = 8.1 Hz, 1H, ArH), 6.90 (d, *J* = 7.9 Hz, 1H, ArH), 5.91 (s, 1H, NH), 2.29–2.20 (m, 2H, CH_2_), 2.06–1.95 (m, 2H, CH_2_), 1.87–1.74 (m, 2H, CH_2_). ^13^C NMR (DMSO-*d*_6_, 100 MHz): *δ* (ppm): 200.3, 199.2, 179.0, 1489, 148.5, 143.0, 138.0, 134.7, 130.0, 125.6, 126.0, 117.8, 116.0, 112.2, 111.0, 90.0, 80.0, 63.3, 60.0, 38.3, 31.0, 22.4.

#### 12-Amino-5-(4-iodophenyl)-7,7-dimethyl-9,10-dioxo-5,6,7,8,9,10-hexahydro-4*b*,9*b*-(epoxyetheno)indeno[1,2-*b*]indole-11-carbonitrile (5f)

Mp: 219–221 °C;^15^ FTIR (KBr) (*v*_max_/cm^−1^): 3403, 3317, 2925, 2191, 1708, 1673,1642, 1593, 1537, 1488, 1457, 1419, 1350, 1264, 1219, 1196, 1146, 1074, 1008, 920, 844, 767, 656; ^1^H NMR (DMSO-*d*_6_, 400 MHz): *δ* (ppm): 8.93 (s, 1H, NH), 8.05–7.98 (m, 2H, ArH), 7.92–7.86 (dd, *J* = 16.8, 7.9 Hz, 2H, ArH), 7.78–7.74 (t, *J* = 7.6 Hz, 1H, ArH), 7.66–7.63 (m, 1H, ArH), 7.09 (s, 1H, ArH), 6.86 (d, *J* = 7.8 Hz, 1H, ArH), 5.92 (s, 1H, NH), 2.57 (d, *J* = 28.9 Hz, 1H, CH), 2.29–2.19 (m, 1H, CH), 1.90 (d, *J* = 15.5 Hz, 1H, CH), 1.77 (d, *J* = 17.2 Hz, 1H, CH), 0.96 (s, 3H, CH_3_), 0.92 (s, 3H, CH_3_). ^13^C NMR (DMSO-*d*_6_, 100 MHz): *δ* (ppm): 200.1, 199.0, 179.3, 149.5, 148.2, 142.5, 138.3, 134.4, 130.0, 125.0, 126.0, 118.2, 115.0, 112.5, 111.3, 90.0, 80.0, 64.3, 60.0, 38.3, 31.2, 22.1.

#### 12-Amino-5-(3-nitrophenyl)-7,7-dimethyl-9,10-dioxo-5,6,7,8,9,10-hexahydro-4*b*,9*b* (epoxyetheno)indeno[1,2-*b*]indole-11-carbonitrile (5g)

Mp: 231–233 °C;^16^ FTIR (KBr) (*v*_max_/cm^−1^): 3401, 3317, 3199, 2924, 2192, 1747, 1707, 1674, 1643, 1592, 1460, 1421, 1351, 1312, 1265, 1220, 1197, 1075, 1012, 921, 841, 765; ^1^H NMR (DMSO-*d*_6_, 400 MHz): *δ* (ppm): 9.10 (s, 1H, NH), 8.40 (d, *J* = 8.4 Hz, 1H, ArH), 8.25 (s, 1H, ArH), 8.05–7.98 (m, 1H, ArH), 7.89 (d, *J* = 7.6 Hz, 1H, ArH), 7.85 (t, *J* = 8.1 Hz, 1H, ArH), 7.74–7.64 (m, 2H, ArH), 6.81 (d, *J* = 7.7 Hz, 1H, ArH), 5.96 (s, 1H, NH), 2.63 (d, *J* = 16.6 Hz, 1H, CH), 2.30 (t, *J* = 15.6 Hz, 1H, CH), 1.88 (dd, *J* = 46.6, 16.4 Hz, 2H, CH_2_), 0.98 (s, 3H, CH_3_), 0.93 (s, 3H, CH_3_).

#### 12-Amino-5-(*P*-tolylphenyl)-7,7-dimethyl-9,10-dioxo-5,6,7,8,9,10-hexahydro-4*b*,9*b*-(epoxyetheno)indeno[1,2-*b*]indole-11-carbonitrile (5h)

Mp: 213–216 °C;^16^ FTIR (KBr) (*v*_max_/cm^−1^): 3402, 3317, 3198, 2925, 2191, 1747, 1707, 1674, 1643, 1593, 1543, 1511, 1461, 1419, 1350, 1312, 1265, 1219, 1196, 1145, 1075, 1012, 921, 843, 765, 657; ^1^H NMR (DMSO-*d*_6_, 400 MHz): *δ* (ppm): 8.82 (s, 1H, NH), 8.05–7.98 (m, 1H, ArH), 7.87 (d, *J* = 7.5, 1H, ArH), 7.74 (t, *J* = 7.5 Hz, 1H, ArH), 7.64 (q, *J* = 7.5, 6.3 Hz, 1H, ArH), 7.34 (d, *J* = 7.9 Hz, 2H, ArH), 7.16 (s, 1H, ArH), 6.86 (d, *J* = 7.8 Hz, 1H, ArH), 5.92 (s, 1H, NH), 2.47 (d, *J* = 9.8 Hz, 1H, CH), 2.40 (s, 3H, CH_3_), 2.29–2.19 (m, 1H, CH), 1.89 (d, *J*=15.5 Hz, 1H, CH), 1.72 (d, *J* = 17.2 Hz, 1H, CH), 0.96 (s, 3H, CH_3_), 0.91 (s, 3H, CH_3_).

#### 12-Amino-5-(4-methoxyphenyl)-7,7-dimethyl-9,10-dioxo-5,6,7,8,9,10-hexahydro-4*b*,9*b*-(epoxyetheno)indeno[1,2-*b*]indole-11-carbonitrile (5i)

Mp: 181–184 °C;^16^ FTIR (KBr) (*v*_max_/cm^−1^): 3374, 3306, 3244, 3195, 2958, 2189, 1748, 1716, 1687, 1660, 1595, 1466, 1422, 1349, 1324, 1292, 1261, 1219, 1163, 1053, 970, 882, 817, 795, 773, 655; ^1^H NMR (DMSO-*d*_6_, 400 MHz): *δ* (ppm): 8.77 (s, 1H, NH), 8.02 (d, *J* = 7.9 Hz, 2H, ArH), 7.87 (d, *J* = 7.7 Hz, 1H, ArH), 7.75 (t, *J* = 7.6 Hz, 1H, ArH), 7.70–7.62 (m, 2H, ArH), 7.08 (d, *J* = 7.8 Hz, 1H, ArH), 6.89 (d, *J* = 7.9 Hz, 1H, ArH), 5.91 (s, 1H, NH), 3.83 (s, 3H, OCH_3_), 2.61 (s, 1H, CH), 2.28–2.18 (m, 1H, CH), 1.89 (d, *J* = 15.7 Hz, 1H, CH), 1.73 (d, *J* = 17.2 Hz, 1H, CH), 0.96 (s, 3H, CH_3_), 0.91 (s, 1H, CH_3_).

#### 12-Amino-5-(4-boromophenyl)-7,7-dimethyl-9,10-dioxo-5,6,7,8,9,10-hexahydro-4*b*,9*b*-(epoxyetheno)indeno[1,2-*b*]indole-11-carbonitrile (5j)

Mp: 227–229 °C;^16^ FTIR (KBr) (*v*_max_/cm^−1^): 3402, 3316, 3190, 2927, 2191, 1747,1707, 1673, 1642, 1594, 1489, 1458, 1420, 1350,1311, 1264, 1219, 1146, 1074, 1011, 920, 870, 840, 766, 656; ^1^H NMR (DMSO-*d*_6_, 400 MHz): *δ* (ppm): 8.95 (s, 1H, NH), 7.87 (d, *J* = 7.7 Hz, 1H, ArH), 7.76 (t, *J* = 7.6 Hz, 1H, ArH), 7.67–7.62 (m, 3H, ArH), 7.32 (s, 2H, ArH), 6.85 (d, *J* = 7.8 Hz, 1H, ArH), 5.93 (s, 1H, NH), 2.51 (d, *J* = 17.3 Hz, 1H, CH), 2.27 (d, *J* = 15.6 Hz, 1H, CH), 1.90 (d, *J* = 15.5 Hz, 1H, CH), 1.74 (d, *J* = 15.6 Hz, 1H, CH), 0.96 (s, 3H, CH_3_), 0.92 (s, 3H, CH_3_).

#### 12-Amino-5-(4-chlorophenyl)-7,7-dimethyl-9,10-dioxo-5,6,7,8,9,10-hexahydro-4*b*,9*b*-(epoxyetheno)indeno[1,2-*b*]indole-11-carbonitrile (5k)

Mp: 222–225 °C;^16^ FTIR (KBr) (*v*_max_/cm^−1^): 3376, 3302,3239, 3106, 2928, 2192, 1746, 1725, 1685, 1660, 1593, 1420, 1340, 1348, 1322, 1259, 1218, 1158, 1050, 770, 725, 650, 616, 551, 503; ^1^H NMR (DMSO-*d*_6_, 400 MHz): *δ* (ppm): 8.95 (s, 1H, NH), 7.87 (d, *J* = 7.7 Hz, 1H, ArH), 7.76 (t, *J* = 7.6 Hz, 1H, ArH), 7.67–7.62 (m, 3H, ArH), 7.32 (s, 2H, ArH), 6.86 (d, 1H, ArH), 5.93 (s, 1H, NH), 2.51 (d, *J* = 15.5 Hz, 1H, CH), 2.27 (d, *J* = 15.6 Hz, 1H), 1.90 (d, *J* = 15.5 Hz, 1H), 1.74 (d, *J* = 15.4 Hz, 1H, CH), 0.96 (s, 3H, CH_3_), 0.92 (s, 3H, CH_3_).

## Results and discussion

### Characterization of M-MNS/CuO

The synthesized nanocatalyst was characterized by XRD, SEM, TEM, EDX, VSM, BET and elemental mapping techniques for their crystalline structure, morphological characteristics, size, elemental composition, magnetic and surface properties.

**Fig. 2:**
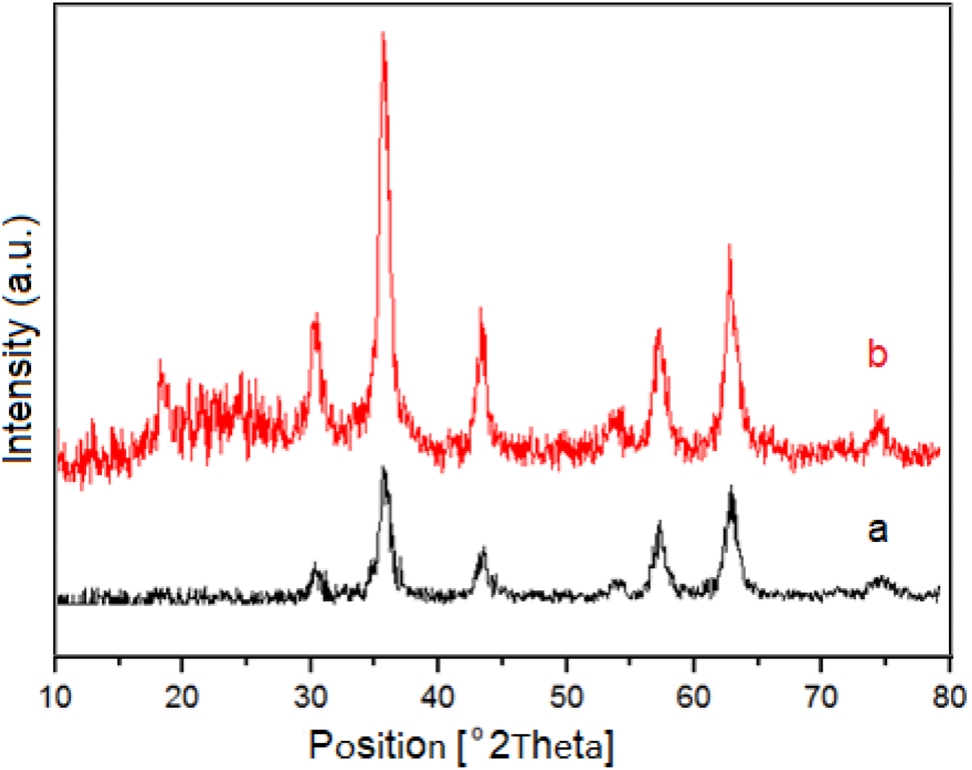
XRD pattern of: (a) NiFe_2_O_4_ and (b) M-MNS/CuO as catalyst.

Powder X-ray diffraction (XRD) patterns was used to investigate the crystalline structure of NiFe_2_O_4_ as magnetic core and M-MNS/CuO (Fig. 2). In XRD pattern of M-MNS/CuO the broad peak at around 2*θ* = 21–26 is concerned to the amorphous structure of silica shell of catalyst.^34^ The other peaks are attributed to the NiFe_2_O_4_ core and CuO NPs. The characteristic peaks located at 2*θ* = 32.58°, 35.47°, and 48.74° are assigned to (110), (002), and (202) plane orientation of CuO nanoparticles which overlapped with some of the main peaks of NiFe_2_O_4_.

**Fig. 3:**
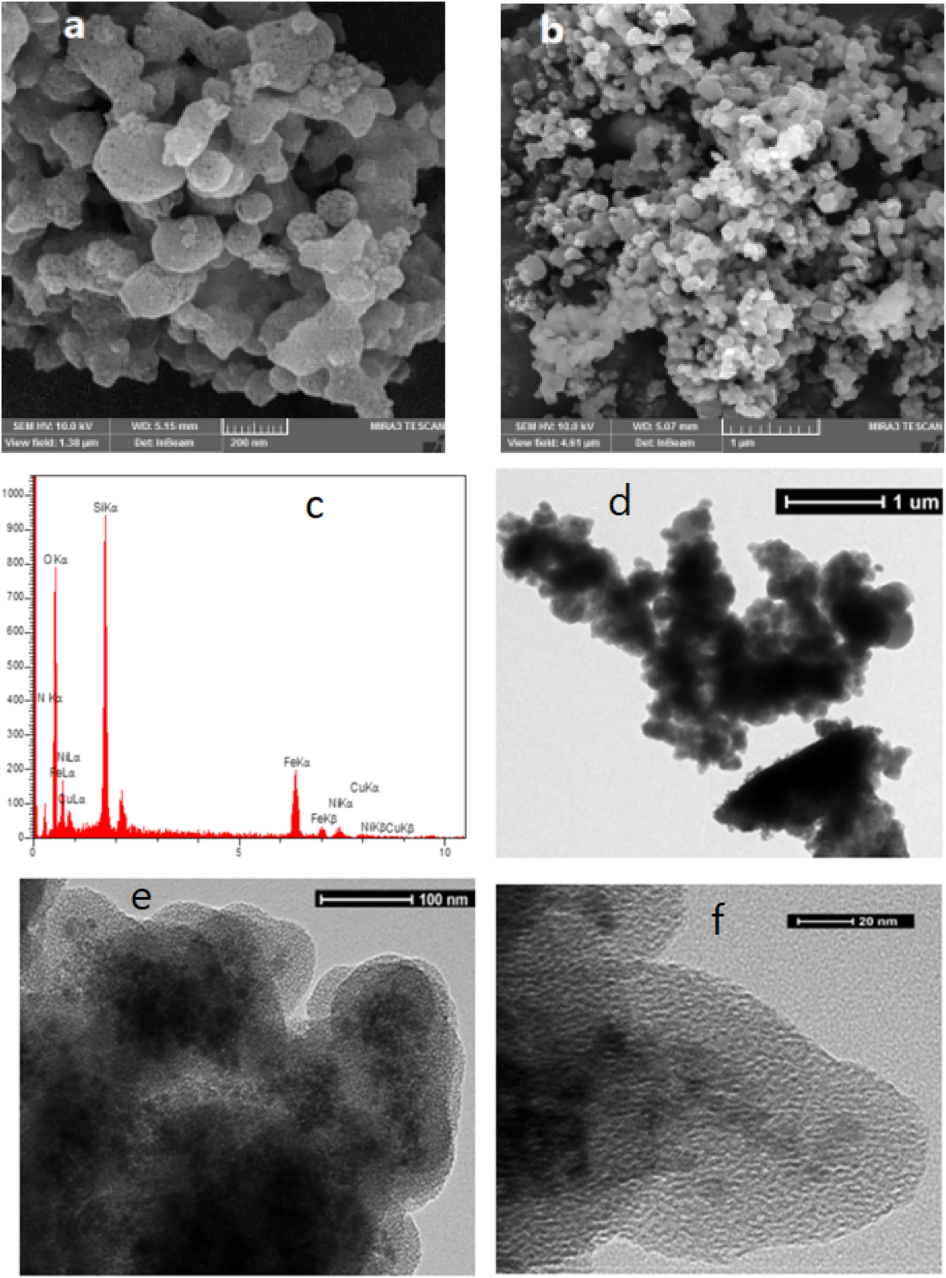
FE-SEM (a, b), EDS (c) and HRTEM of M-MNS/CuO (d–f).

The morphology of the prepared catalyst was investigated by FE-SEM photographs. As can be seen in Fig. 3(a and b), M-MNS/CuO has spherical and amorphous morphologies. The further hydrolysis of silica in the presence of DEA causes the formation of amorphous morphology.

The catalyst composition was evaluated using X-ray energy spectroscopy EDX analysis method (Fig. 3) and the existence of Ni, Fe, N, Si, O and Cu demonstrated the successful preparation of designed catalyst. The core–shell structure of M-MNS/CuO was shown thorough HRTEM (Fig. 3). Magnetic cores trapped in silica are clearly visible.

**Fig. 4:**
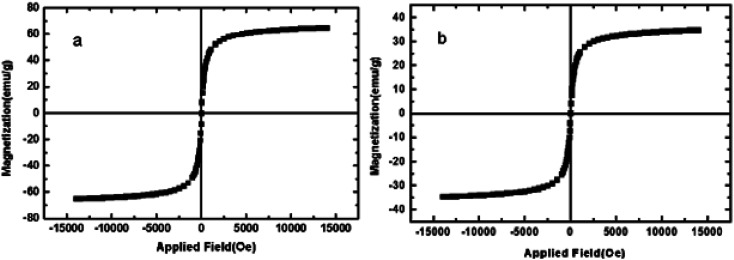
VSM analysis of: NiFe_2_O_4_ NPs (a) and M-MNS/CuO (b).

In VSM analysis, the magnetic property of catalyst (Fig. 4b) is compared with the magnetic NiFe_2_O_4_ core (Fig. 4a). The lower magnetic property of catalyst compare to magnetic behaviour of NiFe_2_O_4_ indicates the coating of NiFe_2_O_4_ nanoparticles by N-doped silica shell which prove the successfully formation of the catalyst core–shell structure.

**Fig. 5:**
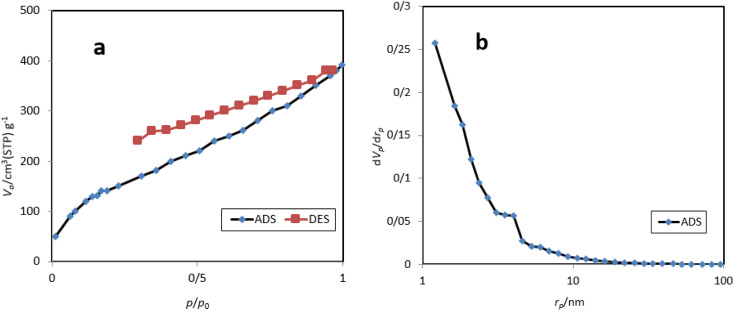
(a) Nitrogen adsorption–desorption isotherms and (b) BJH plots of M-MNS/CuO.

Nitrogen adsorption–desorption isotherms and BJH plots of M-MNS/CuO are shown in Fig. 5. The specific surface area obtained from this analysis is 390 m^2^ g^−1^ which is suitable for the performing chemical reactions on the surface of this catalyst. The total pore volume of the M-MNS/CuO is 0.36 cm^3^ g^−1^ and the mean pore diameter is 3.92 nm that confirms the mesoporous structure of catalyst.

### Optimization of reaction conditions

After preparation and characterization of M-MNS/CuO, the catalyst was applied to synthesis of heterocyclic propellane derivatives. Firstly, to evaluate the optimum reaction conditions, various solvents and different amount of catalyst were tested using a model reaction including malononitrile, ninhydrine, 4-methoxy aniline and dimedone. The results showed the reaction has the best efficiency in water solvent and 0.012 g of catalyst (Table 1).

**Table 1:** Optimization of the solvent and catalyst amount for the synthesis of propellanes^a^

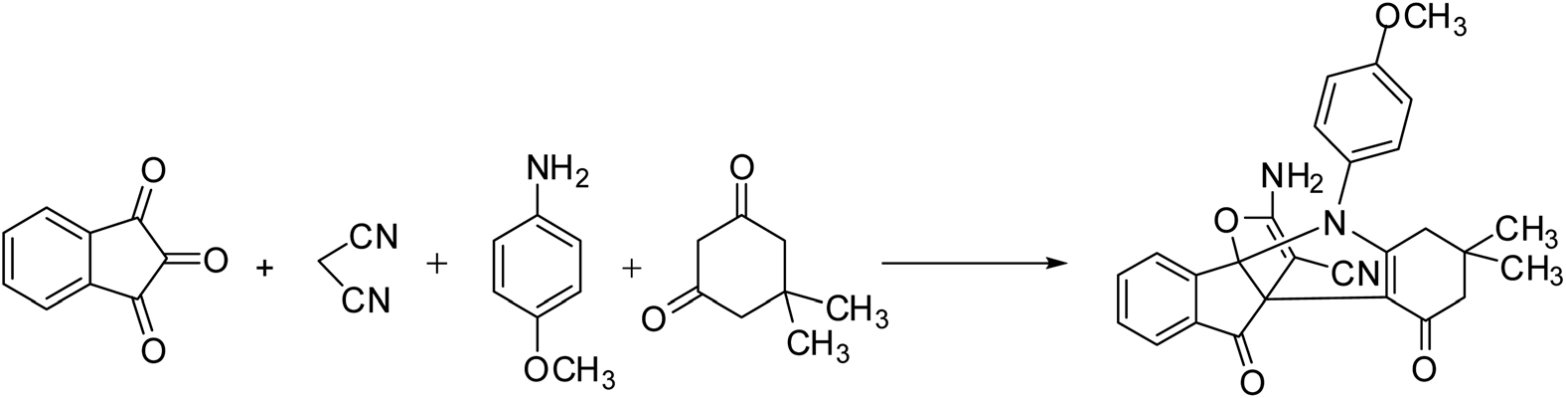				
Entry	Catalyst (g)	Solvent	Time (h)	Yield (%)
1	0.08 g	H_2_O	2.5	60
2	0.01 g	H_2_O	2.5	75
3	0.012 g	H_2_O	2.5	87
4	0.012 g	EtOH	3	82
5	0.012 g	EtOH/H_2_O	2.5	74
6	0.012 g	CH_3_CN	3.5	62
7	0.014 g	PhCH_3_	3.5	65

a Reaction conditions: malononitrile (1 mmol), ninhydrine (1 mmol), 4-methoxy aniline (1 mmol), dimedone (1 mmol), 6 ml solvent in the presence of M-MNS/CuO.

In continue, catalytic activity of M-MNS/CuO was studied for the preparation of some propellane derivatives. Results in Table 2 show that aniline with electron-donating groups increases the yield of products, but the electron withdrawing groups reduces the reaction yield. The obtained products were characterized by FTIR, ^1^H NMR, and ^13^C NMR spectra.

**Table 2:** Investigation of MCNS/CuO performance in the synthesis of propellans^a^

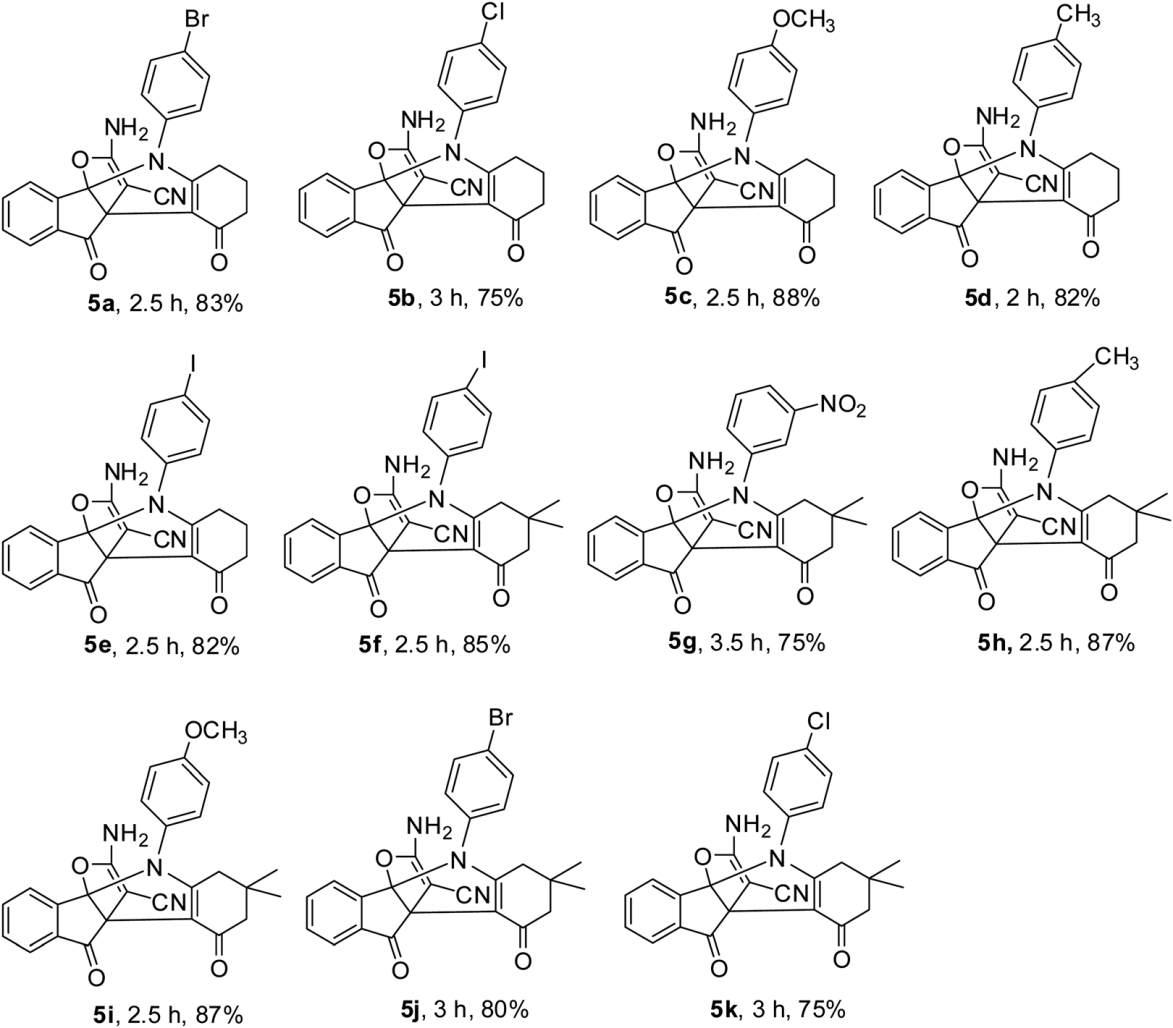

a Reaction conditions: malononitrile (1 mmol), ninhydrine (1 mmol), aniline (1 mmol), β-diketone (1 mmol) in 6 ml H_2_O and 0.012 g M-MNS/CuO at room temperature.

### Proposed mechanism

**Scheme 3:**
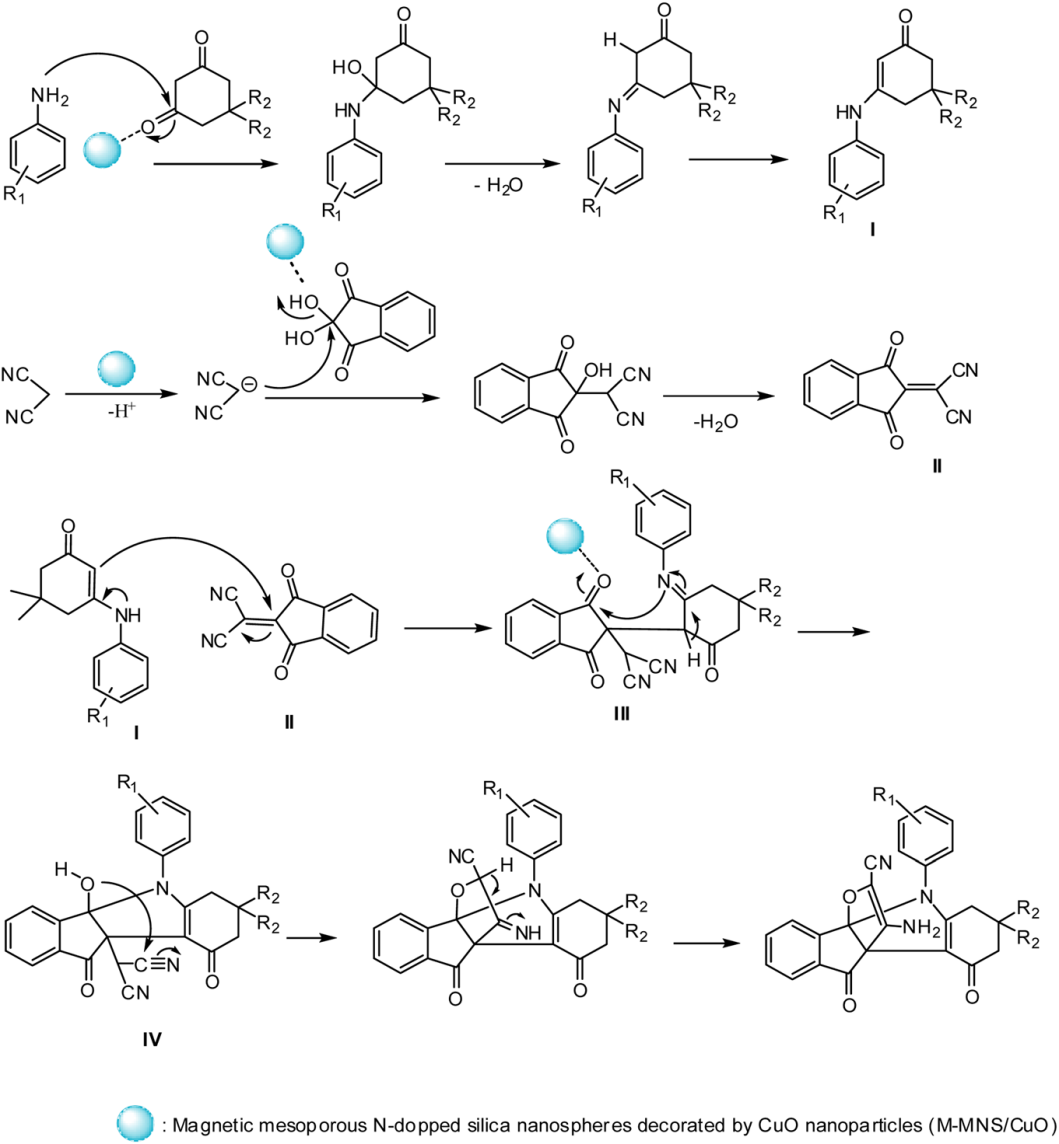
Proposed mechanism for the preparation of [3.3.3] propellane indeno[1,2-*b*] indole in the presence of M-MNS/CuO.

The M-MNS/CuO catalyst has both (Lewis) acidic and basic sites. Nitrogen atoms doped on the surface of the catalyst separated the malononitrile acidic hydrogen, and copper oxide NPs activated the carbonyl groups. As can be seen in Scheme 3, At first, the reaction of aniline and activated diketone follow by water removal and tautomerization, produced intermediate I (which was isolated and characterized with ^1^H NMR). On the other hand, from the reaction of malononitrile and ninhydrin during the Knowenogle reaction in the presence of catalyst, intermediate II was obtained. The reaction of intermediates I and II, follow by internal ring formation, resulted compound IV. The final product is obtained by attacking the hydroxyl group to cyanide and tautomerization.

### Reusability of MCNS/CuO

**Fig. 6:**
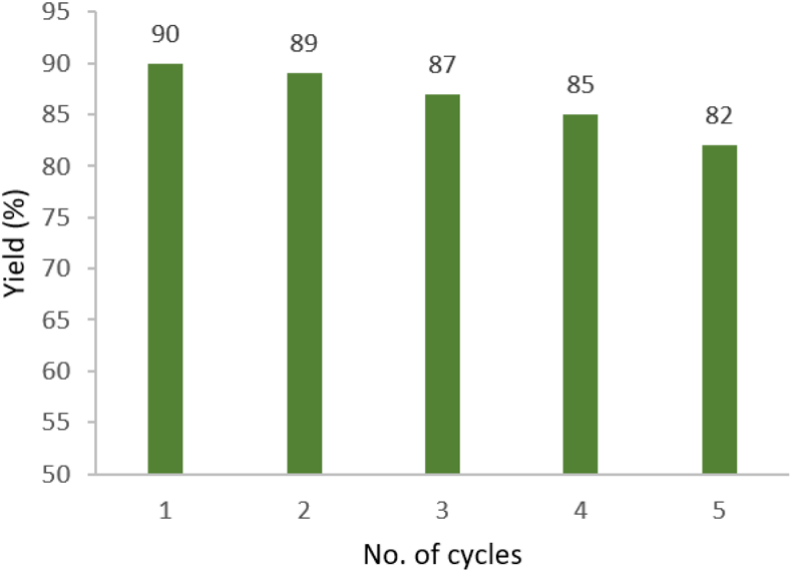
Reusability of M-MNS/CuO.

Reusability of M-MNS/CuO was investigated (using model reaction); to determine the recycling property of catalyst. After separation of the catalyst from the reaction mixture (with a strong magnet), it was washed several times with EtOH and distilled water and dried and used again in another reaction. Obtained results show that there are no considerable changes in catalyst efficiency after 5 cycles (Fig. 6).

## Conclusion

In this work, magnetic core shell N-doped silica decorated by CuO NPs was synthesized successfully and applied as a heterogeneous catalyst for the preparation of some [3.3.3] propellane indeno[1,2-*b*] indole derivatives. Nitrogen doping in silica framework, creates the alkaline property and deposition of CuO NPs gives the acidic property to catalyst. Obtained catalyst is suitable for reactions that require an acidic or basic catalyst.

## Conflicts of interest

There are no conflicts to declare.

## Supplementary Material

RA-012-D2RA06221F-s001
