# Supplementary material for: Modification of magnetic mesoporous N-doped silica nanospheres by CuO NPs: a highly efficient catalyst for the multicomponent synthesis of some propellane indeno indole derivatives
Source: RSC Adv. 2022 Dec 6;12(54):34822–30. doi: 10.1039/d2ra06221f (PMC9724491; doi:10.1039/d2ra06221f)
Supplement: RA-012-D2RA06221F-s001 [file RA-012-D2RA06221F-s001.pdf]

Electronic Supplementary Material (ESI) for RSC Advances.
This journal is © The Royal Society of Chemistry 2022

Supporting information

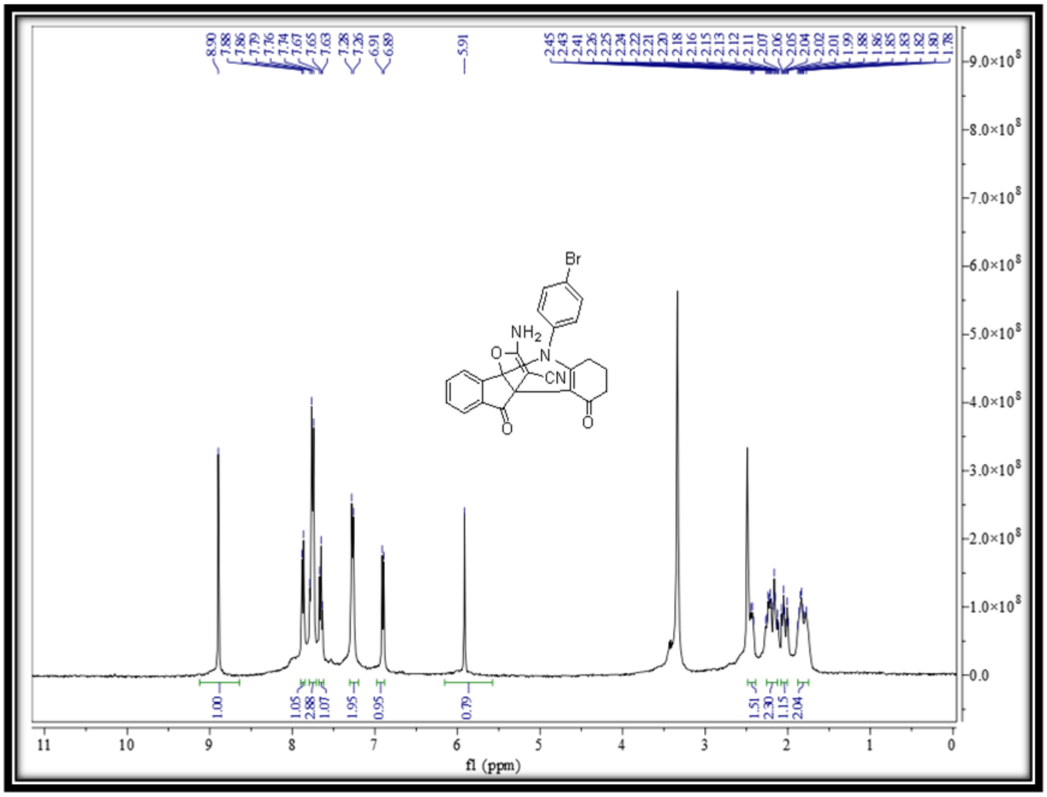

^{1}H NMR of 5a

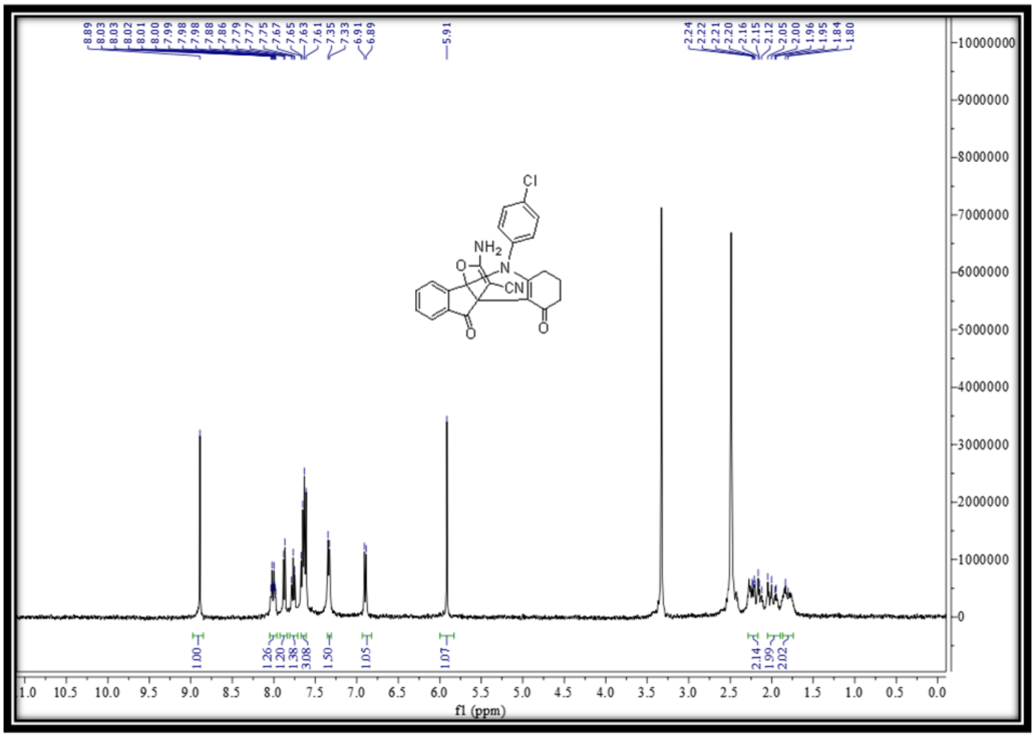

^{1}H NMR of 5b

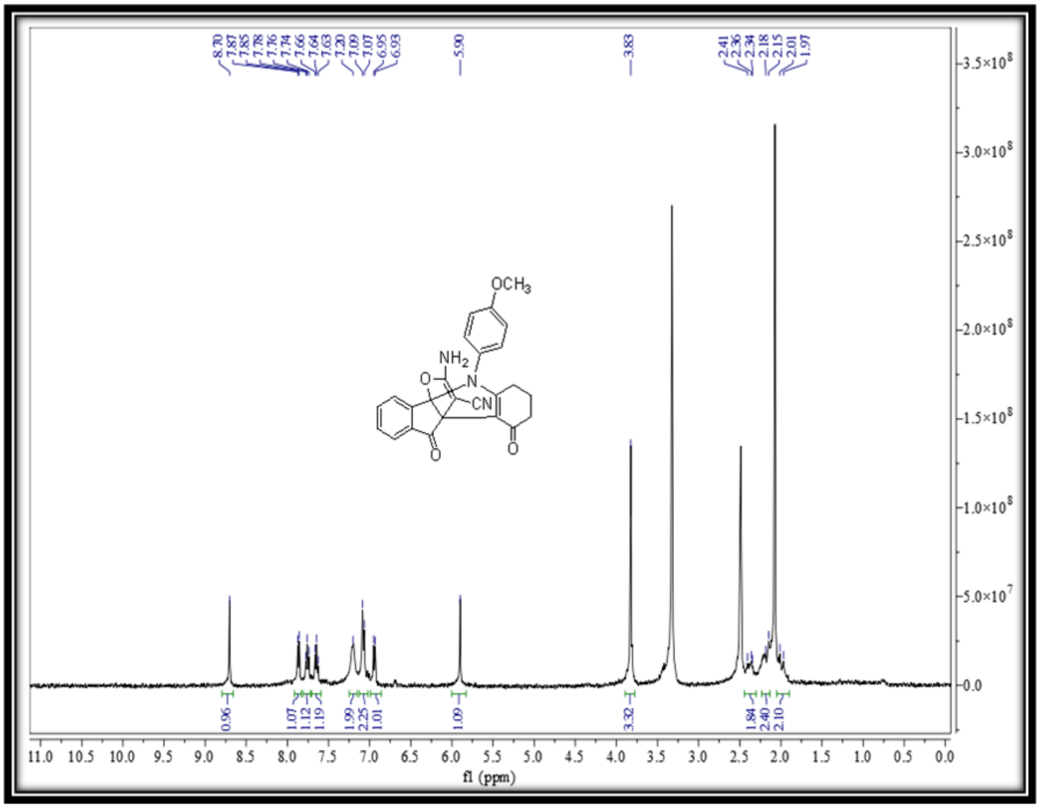

^{1}H NMR of 5c

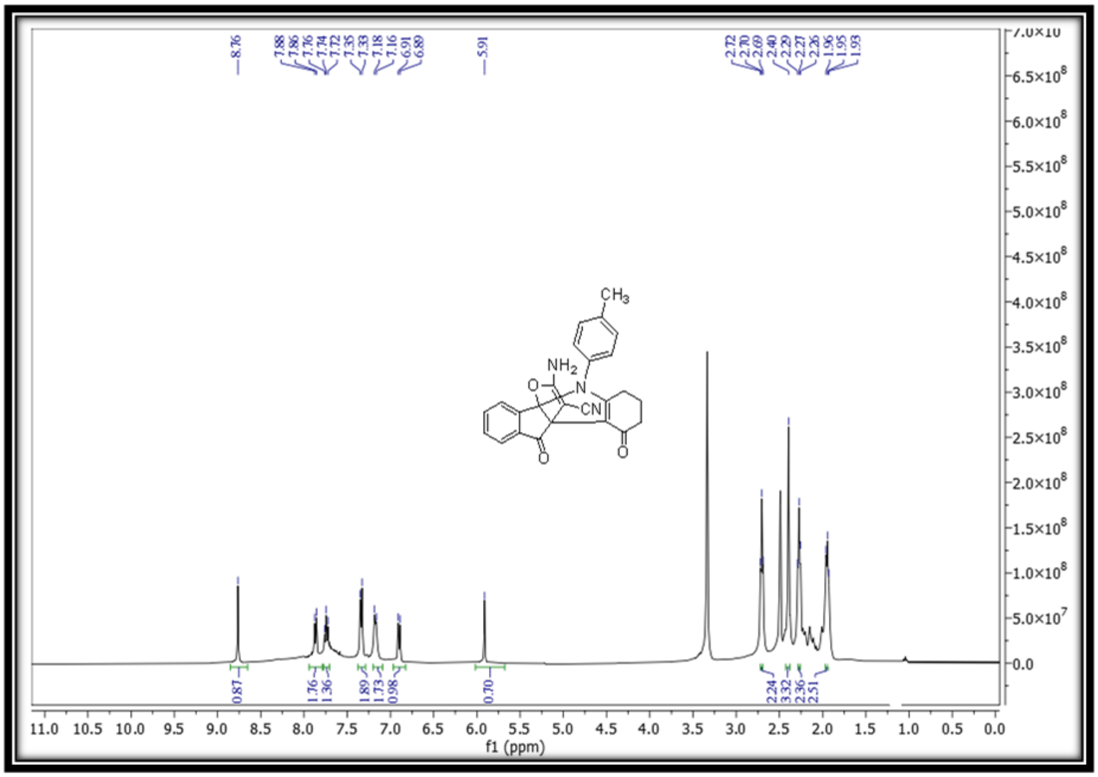

^{1}H NMR of 5d

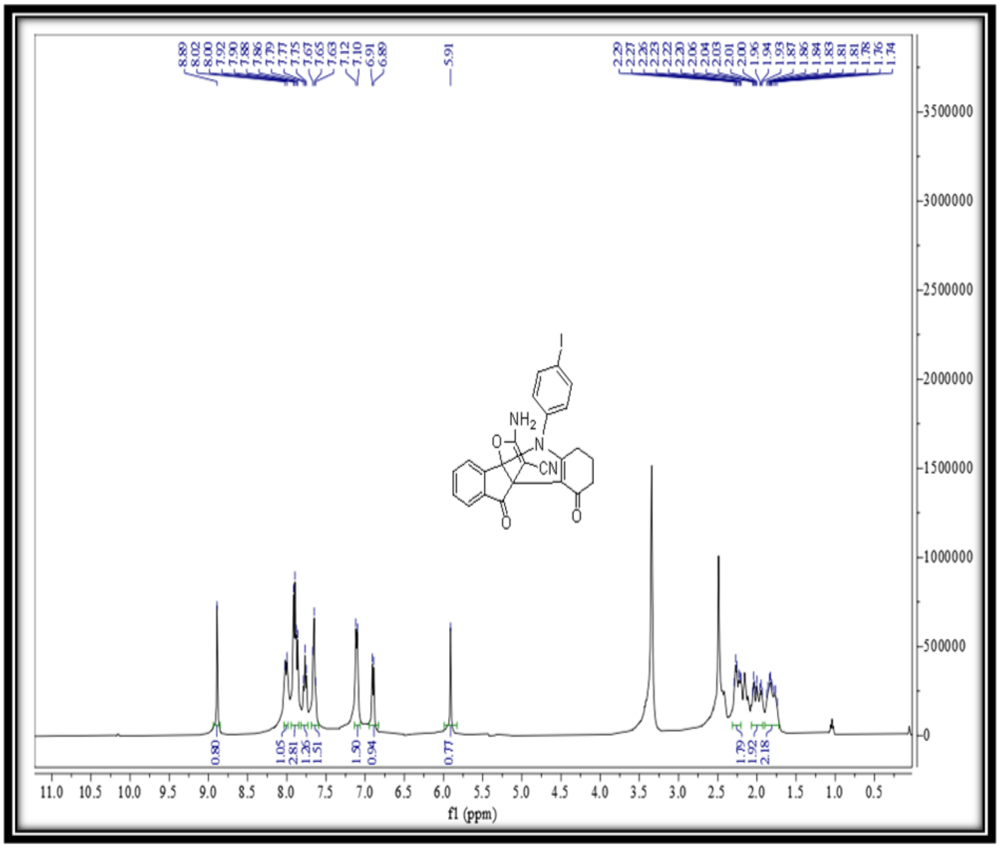

^{1}H NMR of 5e

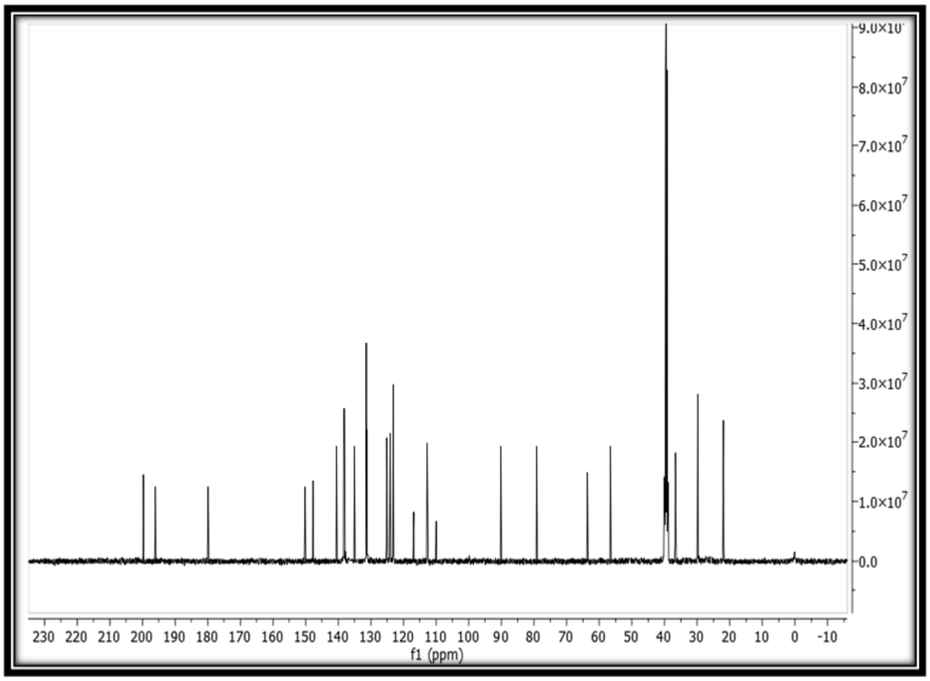

^{13}C NMR of 5e

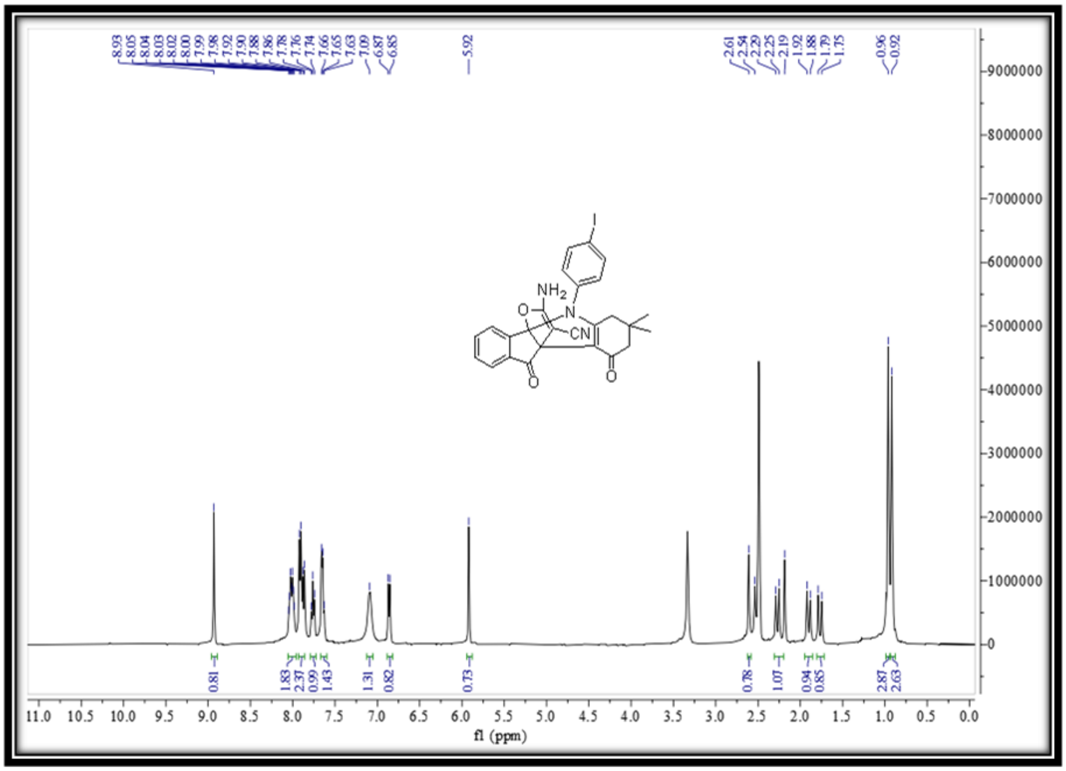

^{1}H NMR of 5f

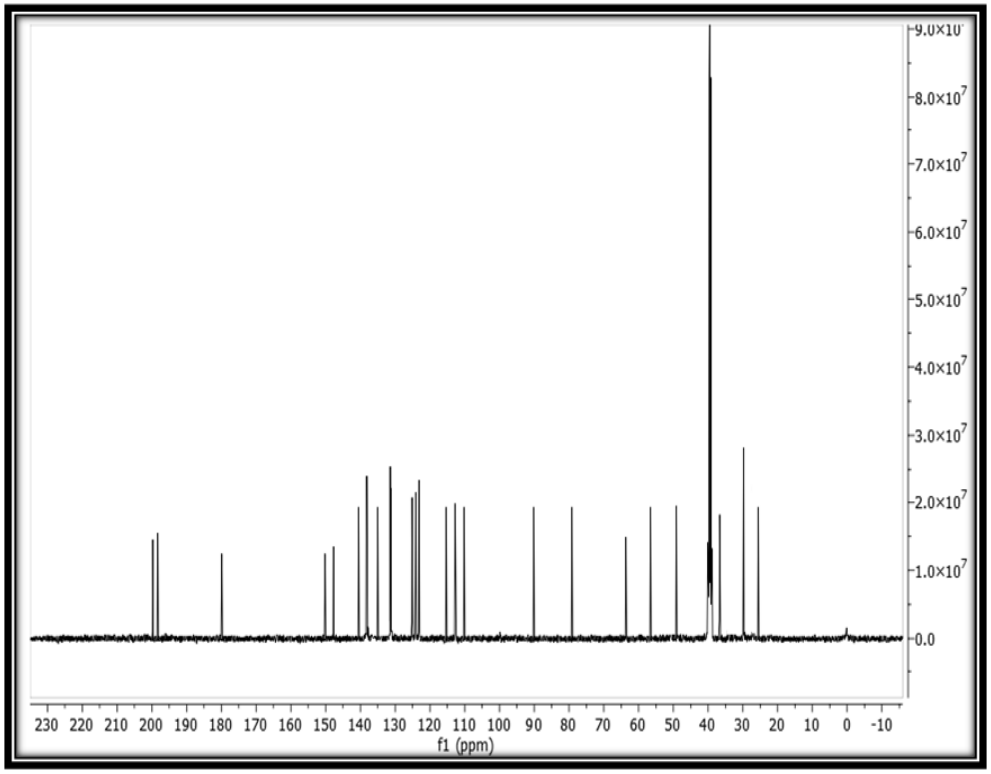

^{13}C NMR o 5f

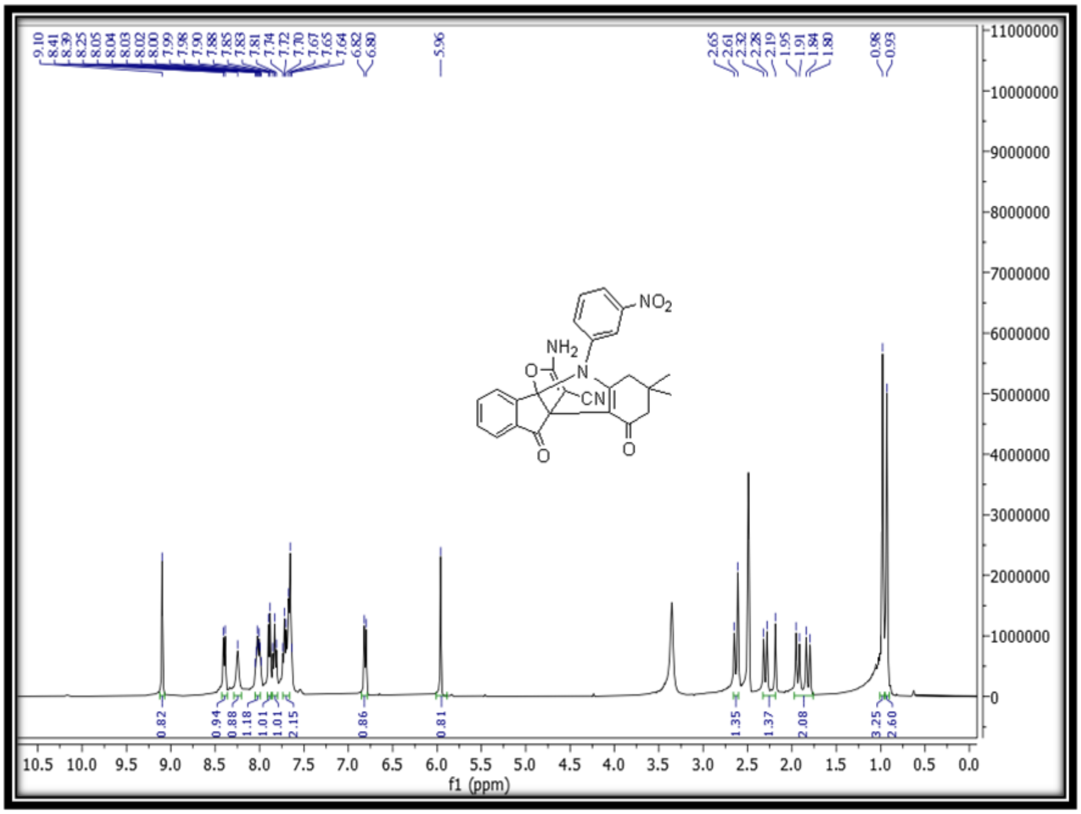

^{1}H NMR of 5g

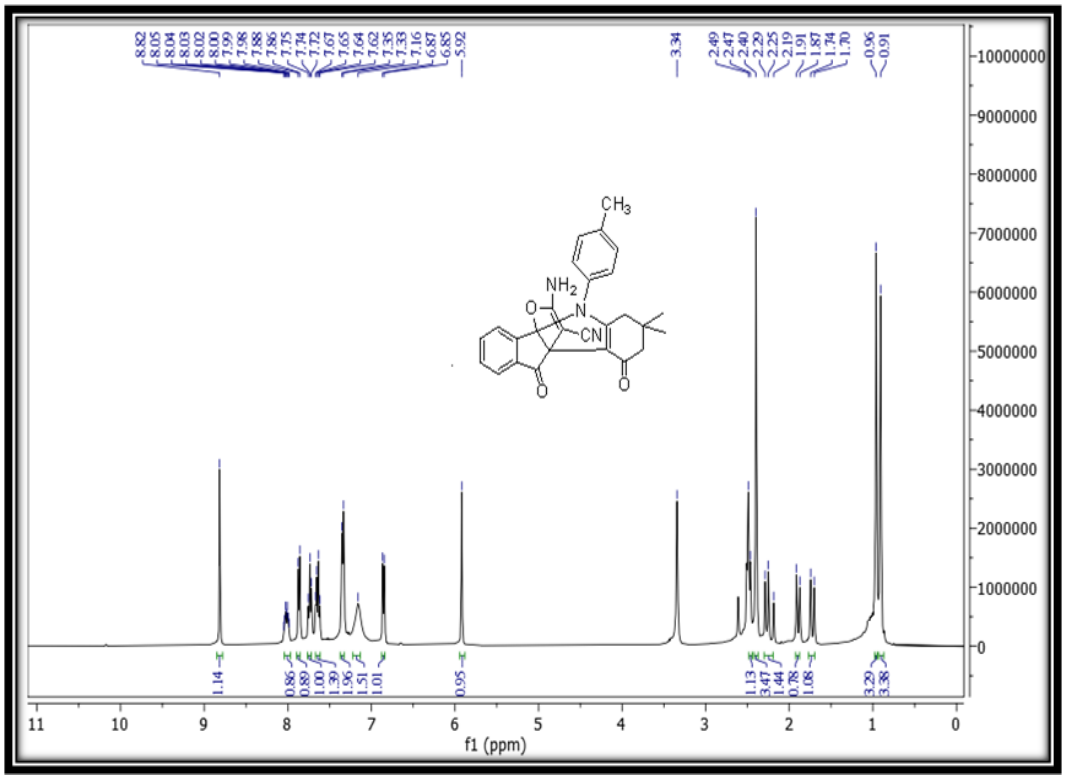

^{1}H NMR of 5h

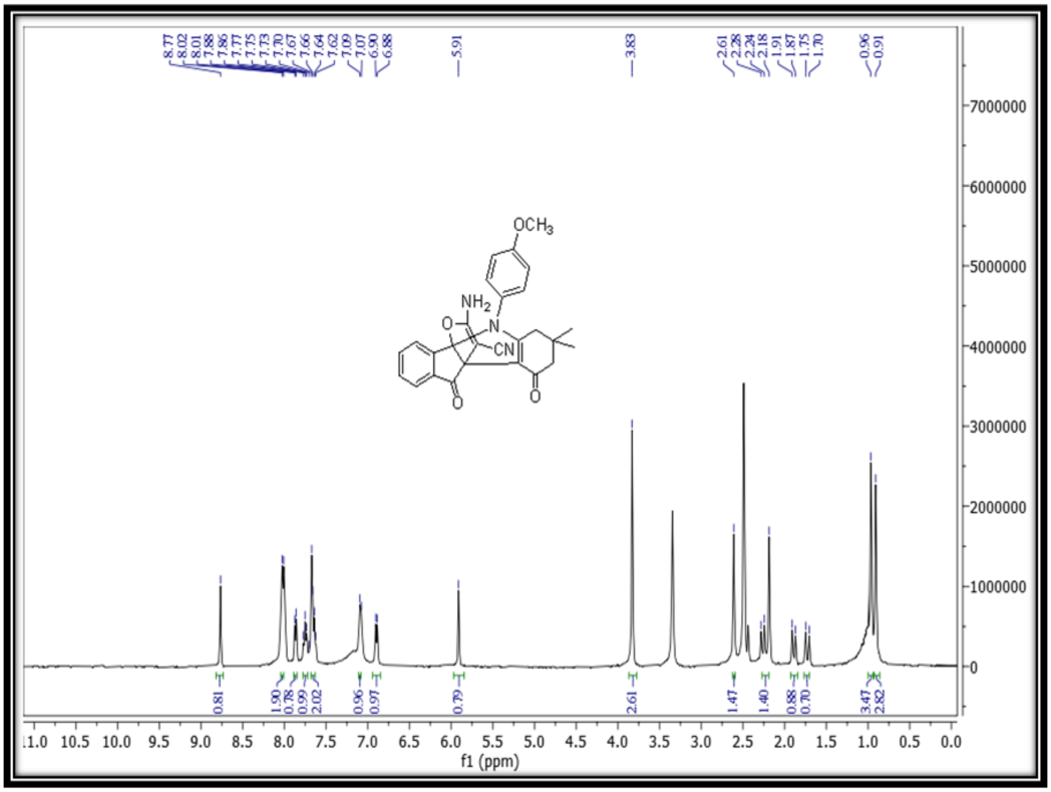

^{1}H NMR of 5i

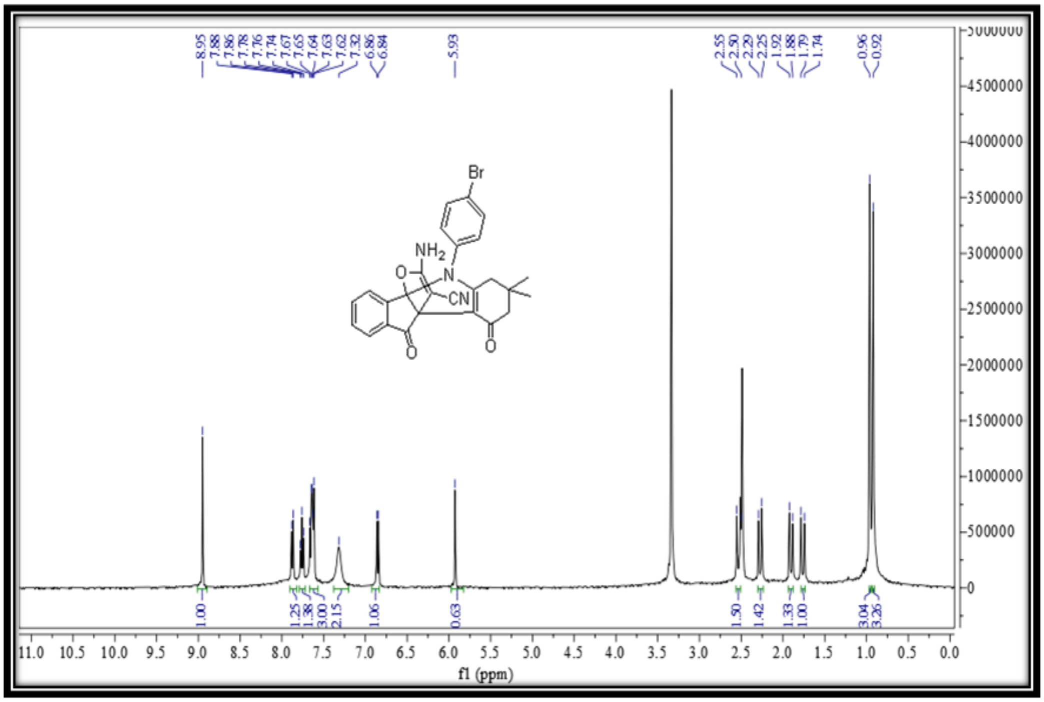

^{1}H NMR of 5j

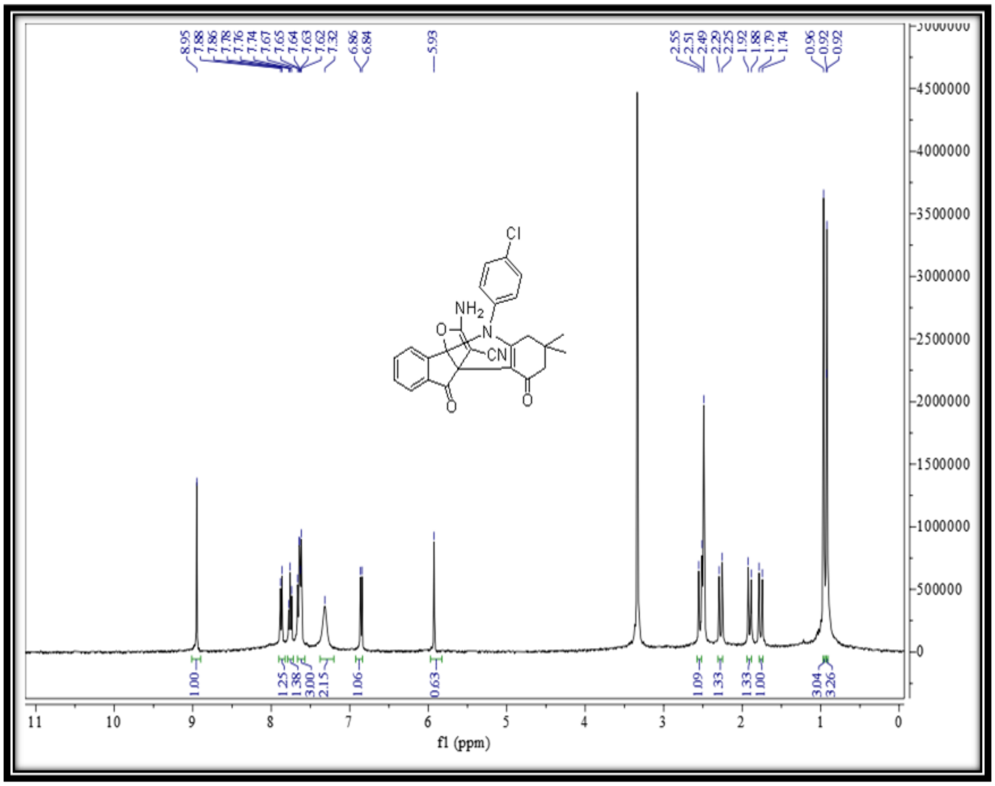

^{1}H NMR of 5k

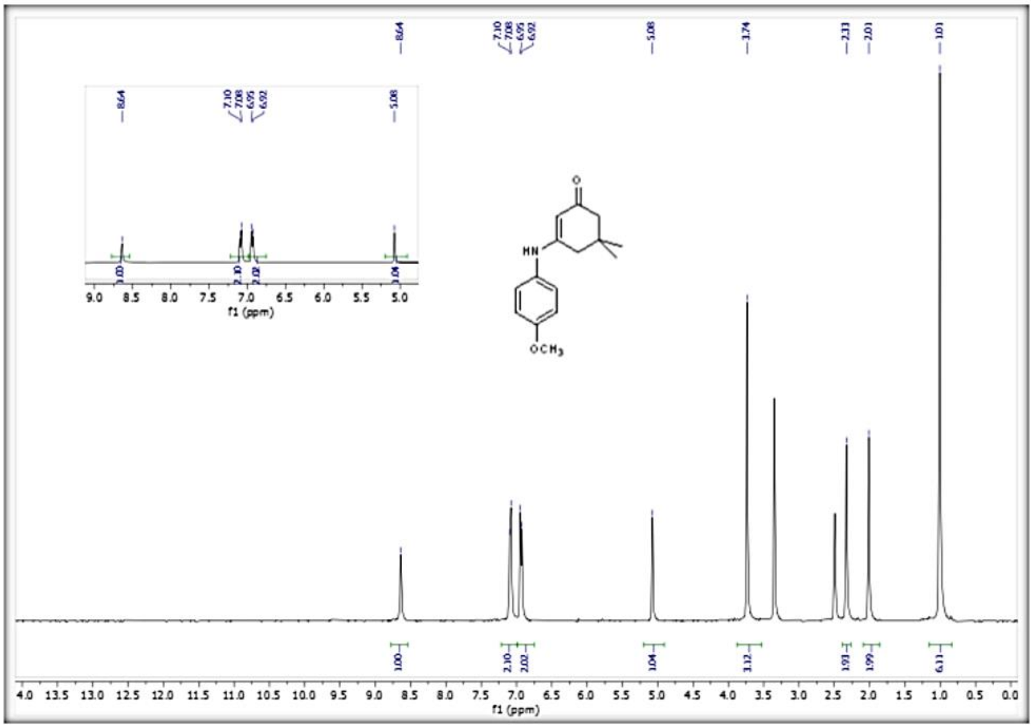

^{1}H NMR of intermediate I
